# Editorial: Inheritance and Improvement of Disease Resistance or Stress Tolerance for *Triticeae* Crops

**DOI:** 10.3389/fgene.2022.877926

**Published:** 2022-03-10

**Authors:** Pengtao Ma, Huagang He, Yi Wang, Yunfeng Xu, Dale Zhang, Cheng Liu

**Affiliations:** ^1^ School of Life Sciences, Yantai University, Yantai, China; ^2^ School of Life Sciences, Jiangsu University, Zhenjiang, China; ^3^ Triticeae Research Institute, Sichuan Agricultural University, Chengdu, China; ^4^ Department of Agronomy, Kansas State University, Manhattan, KS, United States; ^5^ State Key Laboratory of Crop Stress Adaptation and Improvement, College of Agriculture, Henan University, Kaifeng, China; ^6^ Crop Research Institute, Shandong Academy of Agriculture Sciences, Jinan, China

**Keywords:** *Triticeae* crops, stress tolerance, disease resistance, genetic improvement, molecular mechanism editorial on the research topic

## Introduction

*Triticeae* crops are the most important grain crops worldwide. However, they are consistently challenged by biotic and/or abiotic stresses, including pathogens, pests, salt, drought, low and high temperature and heavy metals (Li H. et al., 2019). To combat these threats, the *Triticeae* crops have naturally evolved complex response system to these challenges during their evolutionary history (Langridge and Reynolds, 2021; Tosa 2021). Artificial mutation in the modern period also increased the genetic variation available to cope with these challenges (Krasileva et al., 2017; Chen et al., 2020). In *Triticeae* breeding history, some excellent variations have been selected and used to improve agronomic performance (Li W. et al., 2019). With the development of modern biotechnology and genome/transcriptome sequencing technology, it is expected that many more novel variations will emerge rapidly, and how to identify and use them efficiently in breeding is an important present and future topic. In this topic, recent advances in disease resistance or stress tolerance studies for *Triticeae* crops are presented, in 10 publications including one review and nine research articles, contributed by 72 authors.

## Disease Resistance in *Triticeae* Crops

Powdery mildew is a devastating wheat disease that affect yield and quality. One study identified a broad spectrum powdery mildew resistance gene *PmH4568* in a Chinese wheat cultivar Heng 4,568, which can be used in resistance breeding (Gao et al.). Septoria nodorum blotch (SNB) is a necrotrophic disease of wheat. Francki et al. evaluated the SNB resistance in the glumes of wheat and analyzed its genetic relationship with foliar disease response, and found that glume and foliar response to SNB in wheat is regulated by multiple environment-specific loci which function independently. Wheat root and stem diseases related to soil change have become severe threats to global wheat production. Su et al. summarized the genetics of resistance to three related diseases, including common root rot, fusarium crown rot and sharp eyespot.

## Abiotic Stresses in *Triticeae* Crops

Soil salinization is one of the major abiotic stresses that affect the wheat yield and quality. He et al. identified eight salt-tolerance-related miRNAs and their corresponding 11 target mRNAs that are useful to develop genetically improved salt-tolerant wheat varieties. Tong et al. screened salt-tolerant *Thinopyrum ponticum* under two coastal region salinity stress levels which can be used as excellent germplasm to develop salt-tolerant cultivars. In wheat drought stress, chlorophyll content of the flag leaf is an important trait for drought tolerance. Yang et al. identified several genetic loci affecting flag leaf chlorophyll under different water regimes. In wheat, autophagy is involved in the regulation of variousbiotic and abiotic stresses. Li et al. analyzed the programmed degradation of pericarp cells in wheat grains depends on autophagy.

## Evolution of Gene Families Related to Biotic and Abiotic Stresses in *Triticeae* Crops

Nucleotide binding site (NBS)-leucinerich repeat (LRR) receptor (NBS-LRR, also termed as NLR) is a large and one of the most important gene family against wheat disease. Li et al. and Qian et al. identifiedand characterized the NBS-LRR genes in genome of barely and *Secale cereale*, respectively, which are both important material for the molecular breeding of other *Triticeae* crops. Plant heat shock factor (HSF) is another important gene family against external biotic and abiotic stresses. Li et al. compared the HSF genes from *Secale cereale* and its *Triticeae* relatives, and the results revealed ancient and recent gene expansions of this gene family.
